# Multilocus Sequence Genotype Heterogeneity in *Streptococcus uberis* Isolated from Bovine Mastitis in the Czech Republic

**DOI:** 10.3390/ani12182327

**Published:** 2022-09-07

**Authors:** Monika Zouharova, Katerina Nedbalcova, Natalie Kralova, Petr Slama, Katarina Matiaskova, Jan Matiasovic

**Affiliations:** 1 Department of Infectious Diseases and Preventive Medicine, Veterinary Research Institute, Hudcova 296/70, 621 00 Brno, Czech Republic; 2 Department of Experimental Biology, Faculty of Science, Masaryk University, Kamenice 753/5, 625 00 Brno, Czech Republic; 3 Laboratory of Animal Immunology and Biotechnology, Department of Animal Morphology, Physiology and Genetics, Faculty of AgriSciences, Mendel University in Brno, Zemedelska 1, 613 00 Brno, Czech Republic

**Keywords:** intramammary infection, MLST, antimicrobial resistance, virulence factors, cows

## Abstract

**Simple Summary:**

Bovine mastitis is a serious problem for dairy farmers, resulting in great economic losses. A large number of antimicrobials are used to treat mastitis, contributing to the spread of resistance. *Streptococcus uberis* is an important environmental pathogen responsible for a significant proportion of subclinical (asymptomatic) and clinical intramammary infections in many countries. This pathogen is present in the environment of cows, colonising multiple body sites of the cow, including the mammary gland. Isolates may produce virulence factors that enable the bacteria to infect the mammary gland, resist the defence mechanisms of the mammary gland, and persist inside the gland. *S. uberis* isolates differ in virulence and the level of antimicrobial resistance, posing a challenge to controlling *S. uberis* infection. Therefore, it is necessary to study the biology and genetics of this pathogen to be able to help farmers and veterinarians to implement effective targeted measures against *S. uberis* mastitis.

**Abstract:**

The ubiquitous occurrence and high heterogeneity of *Streptococcus uberis* strains cause difficulties in the development and implementation of effective control strategies in dairy herds. In this study, *S. uberis* strains from 74 farms, obtained predominantly from subclinical, acute, and chronic recurrent mastitis, as well as from udder surface swabs and milk from healthy udders, were analysed for their genetic diversity using multilocus sequence typing (MLST). Isolates were tested for the presence of the genes encoding the virulence factors using polymerase chain reaction. Antibiotic susceptibility testing was performed using a microdilution assay including 14 antimicrobials. The virulence profiles and antimicrobial (AMR) profiles of the isolates were assembled and the overall heterogeneity was evaluated. Among the 124 isolates, 89 MLST genotypes, 7 different virulence profiles, and 12 AMR profiles were identified. The large number of different MLST allelic profiles in this study points to the high heterogeneity of strains in dairy herds in the Czech Republic. Isolates of a certain MLST genotype may possess a different set of virulence factor genes. We detected up to three different resistance profiles within a single MLST genotype. The results of our study showed that fully susceptible isolates coexisted with resistant or even multiresistant isolates in the same herd. Multiple genotypes within a herd were detected on many farms (up to seven MLST genotypes and four AMR profiles in one herd). This heterogenic population structure might suggest that environmental transmission is the predominant route of infection in herds in the Czech Republic.

## 1. Introduction

Mastitis causes great economic losses in dairy farming worldwide due to reduced milk production and quality, treatment costs, or the culling of animals suffering from chronic and persistent mastitis. Mastitis, including its prevention, is the most common indication for the use of antimicrobials in dairy cows, contributing to the spread of resistance.

One of the most common mammary pathogens is *S. uberis*. The frequent occurrence of mastitis caused by this bacterium has several causes. *S. uberis* is a ubiquitous bacterium in the environment of cows, especially in areas where cows congregate and rest, and can also be isolated from various parts of the cow’s body. The place most at risk is the waiting area in front of the milking parlour because of the increased amount of faeces present here. The teat canal is thus constantly exposed to this bacterium. In addition, the teat canal is open at this time and milk leakage can contaminate the area with *S. uberis*, allowing the pathogen to easily penetrate the healthy mammary gland [1].

*S. uberis* is predominantly spread as an environmental pathogen, but some authors have described the possibility of contagious transmission [2,3]. Many known and putative virulence factors have been described, and it has been suggested that their expression varies from one strain to the other [4]. Thus, different *S. uberis* isolates can differ in the transmission mode and also in the virulence mechanism and level of virulence. Even within one farm, a wide range of genetic variants of *S. uberis* isolates can be detected [5]. Some of them are able to infect the mammary gland from the environment and cause transient infections [6]. Some strains may persist in the mammary gland over several lactations and cause chronic recurrent infections, whereas others are unable to overcome the defence mechanisms of the teat canal and the mammary gland [7,8].

It has also been shown that some strains are more resistant to antibiotic treatment than other strains, although they are equally sensitive to the antimicrobials in the laboratory [9,10]. This is possibly due to their ability to form biofilms and penetrate the epithelial cells of the mammary gland. This huge heterogeneity, even within a single herd, is the reason for the difficulty in implementing effective measures against *S. uberis* mastitis.

Since the mid-2000s, multilocus sequence typing (MLST) has emerged as an effective method for genotyping pathogens and is widely implemented in research to determine genetic diversity and understand the epidemiology of *S. uberis* [11,12,13]. The MLST scheme, which is widely used today, was originally developed by Coffey et al. in 2006 [14]. It assigns sequence types (STs) to *S. uberis* isolates based on the allelic profiles of its seven house-keeping genes, which were selected due to their low propensity towards undergoing mutations: carbamate kinase (*arcC*), D-alanine-D-alanine ligase (*ddl*), glucose kinase (gki), transketolase (*recP*), thymidine kinase (*tdk*), triosephosphate isomerase (*tpi*), and acetyl-coA acetyltransferase (*yqiL*) [12,14]. The advantage of this method is the database available on the internet (https://pubmlst.org/organisms/streptococcus-uberis) (accessed on 10 May 2022), which enables the comparisons of isolates worldwide.

The aim of this study was to evaluate the occurrence of the genetic types of *S. uberis* determined by MLST in farms in the Czech Republic, the heterogeneity of *S. uberis* strains between herds and within herds, and the heterogeneity of the strains within the same MLST genotypes.

## 2. Materials and Methods

A total of 124 *S. uberis* isolates originating from 74 dairy farms collected between 2020 and 2021 were included in the study.

### 2.1. Bacterial Sampling

A total of 124 strains of *S. uberis* isolated from subclinical (according to the high number of somatic cells found in the production control programmes, with a cut-off of 400,000 cells per mL) and clinical cow mastitis were used in this study. Swab samples were also taken from the udder surface and several *S. uberis* isolates were cultivated from milk from healthy mammary glands. The samples were collected from 74 different farms in the Czech Republic between 2020 and 2021. After sampling, the milk was kept in containers at 6–8 °C and delivered to the laboratory within 4 h or it was frozen at −18 °C and delivered within one week.

### 2.2. Bacterial Isolation and Identification

Ten microliters of milk samples and/or the swab samples were plated onto Columbia agar (Oxoid, Basingstoke, UK) supplemented with 5% defibrinated sheep blood and cultivated at 37 °C for 24 h. The isolates were assessed based on the colony appearance, Gram stain reaction, and catalase test, and subsequently identified by the phenotypic molecular method using a MALDI-TOF-MS mass detector (Bruker Daltonics GmbH, Bremen, Germany). Subsequently, the strain was confirmed by the detection of the *S. uberis*-specific 16S rRNA gene by polymerase chain reaction (PCR) (Table 1).

### 2.3. Virulence Factors Determination

The regions in the virulence-associated genes and *S. uberis*-specific gene were amplified using three multiplex PCRs: (1) *hasA, hasB, hasC, and sua*; (2) *cfu* and the *S. uberis*-specific 16S rRNA gene; (3) *pauA/skc, gapC,* and *oppF*. The multiplex PCRs were previously optimised for the detection of each set of genes. A few colonies of a pure bacterial culture were resuspended in 50 μL of sterile distilled water. The suspension was incubated for 10 min at 100 °C and centrifuged for 10 min at 10,000× *g*. The supernatant was used in the PCR reaction as the template DNA. The 20 μL reaction mixture contained 10 μL of a HotStarTaq Plus Master Mix 2×, 1 μL of primers (10 pmol/μL) (primer sequences and their product size are shown in Table 1), 2 μL of a CoralLoad Concentrate 10× (Qiagen, Hilden, Germany), 4 μL of DNase-free water, and 2 μL of DNA. The cycling conditions were as follows: initial denaturation at 95 °C for 5 min, followed by 30 cycles of denaturation at 94 °C for 30 s, annealing at 55 °C for 90 s, extension at 72 °C for 90 s, and final extension at 72 °C for 10 min. Ten microliters of the PCR product were electrophoresed on a 2% agarose gel stained with ethidium bromide (Sigma Aldrich, St. Louis, MO, USA) and the PCR products were visualised under ultraviolet light. Isolates in which a particular gene had previously been detected in a simplex PCR reaction were used as a positive control. A positive control for each gene detected was included in each run.

### 2.4. Antimicrobial Susceptibility Testing

Antimicrobial susceptibility testing (AST) of fourteen selected antimicrobials was performed by the determination of the minimum inhibitory concentration (MIC) using the microdilution method according to the internationally recognized methodology of the Clinical and Laboratory Standards Institute [20,21]. The MICs were determined using diagnostic kits manufactured by the co-authors at the Veterinary Research Institute in Brno, the Czech Republic. The growth medium for the dilution of the antimicrobials was Mueller Hinton Broth (BD Difco, Franklin Lakes, United Kingdom) with the addition of 4% Lysed Horse Blood (Labmediaservis, Jaroměř, Czech Republic). The quality of the examination was evaluated by parallel examination of the control reference strain *Streptococcus pneumoniae* ATCC 49619 [21].

The antimicrobials used for AST (Discovery Fine Chemicals Limited, Wimborne, United Kingdom) represented 9 antimicrobial groups: lincosamides (clindamycin, pirlimycin), aminoglycosides (gentamicin, streptomycin), macrolides (erythromycin), sulfonamides (sulfamethoxazole with trimethoprim), tetracyclines (tetracycline), ansamycins (rifampin), and three groups of penicillins: (1) narrow and broad-spectrum, penicillinase-sensitive (penicillin, ampicillin), (2) penicillins with beta-lactamase inhibitors (amoxicillin with clavulanic acid), and (3) cephalosporins (cephalexin–1st generation, ceftiofur–3rd generation, cefquinome–4th generation)

Isolates were categorized as susceptible, intermediate, or resistant using the clinical breakpoints published in the CLSI documents [21], the European Committee on Antimicrobial Susceptibility Testing–Breakpoint tables for bacteria [22], and the Comité de l’Antibiogramme de la Société Francaise de Microbiologie–Recommandations vétérinaires 2018 [23]. A multidrug-resistant isolate was defined as an isolate resistant to at least one substance from three or more antimicrobial groups [24].

### 2.5. Multilocus Sequence Typing

Multilocus sequence typing was performed according to a previously published method [14] with some modifications. PCR for each gene (*arcC*, *ddl*, *gki*, *recP*, *tdk*, *tpi*, and *ygiL*) was performed separately in a total volume of 20 µL consisting of 10 µL of PCR Master Mix 2× (Thermo Fisher Scientific Baltics, Vilnius, Lithuania), 1 µL of primers (final concentration 0.2 µM), 6 µL of DNase-free water, and 2 µL of DNA. PCR conditions were as follows: initial denaturation at 95 °C for 5 min, followed by 35 cycles of denaturation at 94 °C for 45 s, annealing at 55 °C (for *gki*, *tdk*, *arcC*, *ygiL*) or 60 °C (for *recP* and *tpi*) or 65 °C (for *ddl*) for 1 min, elongation at 72 °C for 1 min, and final elongation at 72 °C for 8 min. PCR products were verified by gel electrophoresis and subsequently purified by a column kit Expin Combo GP (GeneAll Biotechnology Co., LTD, Seoul, South Korea).

PCR products of the seven genes were sequenced by the Sanger sequencing method (Eurofins Genomics, Cologne, Germany) in forward and reverse directions. The data from sequenation in FASTA format were directly entered into the PubMLST database (https://pubmlst.org/organisms/streptococcus-uberis) (accessed on 10 May 2022) to identify allelic matches. Each isolate was defined by an allelic profile, which corresponds to the allele numbers at the seven loci in the order *arcC*, *ddl*, *gki*, *recP*, *tdk*, *tpi*, and *yqiL*. According to the combination of alleles, the sequence types (STs) were determined. Unknown allelic profiles were submitted to the database and new STs were generated (in profiles that occurred more than once or showed different virulence profiles) (see Table 2).

## 3. Results

### 3.1. MLST and MLST Genotyping

Out of 124 S. uberis isolates from 74 farms, MLST analysis revealed 89 MLST allelic profiles (genotypes), of which only 21 (23.6%) could be assigned to known STs according to the database. The most common ST was 1135, detected in nine isolates from seven farms, followed by ST 307, which was detected in nine isolates from six farms. Other types that were detected multiple times were ST 1436, ST 316, ST 855, ST 876, and ST 1437. Each of these types was detected in three farms. ST 877, ST 1438, ST 1439, and ST 1440 were each detected on two farms. All other types were detected only on one farm. See Table 2 for a summary.

### 3.2. Virulence Profiling

The virulence profile was determined by combining ten virulence-associated genes. 106 isolates (85.5%) belonged to the profile hasA+, hasB+, hasC+, sua+, cfu-, pauA/skc+, gapC+, and oppF+, thus we called it the “common” profile (Table 2). Eighteen isolates from seventeen farms showed different profiles (Table 2). The genes hasC, sua, gapC, and oppF were detected in all isolates (100%), and gene pauA/skc was detected in all but three isolates (97.6%). The cfu gene was found only in 11 (8.9%) isolates. The PCR products for hasA and hasB were not detected in 11 (8.9%) isolates; these two genes always occurred together.

### 3.3. Antimicrobial Resistance Profiling

Based on the testing of the susceptibility to 14 antimicrobials, the profiles of phenotypic antimicrobial resistance (AMR) were assembled. For simplicity and clarity, we designated the individual AMR profiles with letters of the alphabet. Most isolates (n = 42; 34%) were susceptible or intermediately susceptible to all tested antimicrobials (profile designated with the letter S in Table 2). The other two most common AMR profiles were profile A (n = 37; 29.8%), which was resistant to tetracycline (TET), and profile B (n = 26; 21 %), which was resistant to TET, streptomycin (STR), and clindamycin (CLI). Other profiles occurred with a significantly lower frequency: profile E (n = 4; 3.2%) was resistant to STR, CLI, and pirlimycin (PIR); profile D (n = 3; 2.4%) was resistant to STR and CLI; profile H (n = 3; 2.4%) was resistant to TET and erythromycin (ERY); profile C (n = 2; 1.6%) was resistant to TET and STR; profile L (n = 2; 1.6%) was resistant to TET, CLI, PIR, and ERY; profile F (n = 1; 0.8%) was resistant to STR and rifampicin (RIF); profile G (n = 1; 0.8%) was resistant to TET and CLI; profile I (n = 1; 0.8%) was resistant to TET, STR, CLI, PIR, and ERY; profile J (n = 1; 0.8%) was resistant to STR; and profile K (n = 1; 0.8%) was resistant to TET, CLI, and ERY.

### 3.4. Distribution of AMR Profiles and Virulence Profiles in MLST Genotypes

Isolates of ST 307 showed two different virulence profiles in addition to the common virulence profile and they were positive for the cfu gene. In the other cases, no different virulence profiles within the same ST were observed (Table 2).

The greatest variability within a single MLST genotype was observed in ST 307. Isolates from this genotype were detected in six farms and showed two virulence profiles and three AMR profiles (see Table 2). These isolates thus created five variations within ST 307. In two farms, two different AMR profiles of S. uberis ST 307 within the same herd were detected at the same sampling time (profile S and A in one herd; profile S and D in the other herd).

ST 1135 was detected in seven farms; the virulence profile was the same in all the farms, but the isolates showed three different AMR profiles and thus three variations within ST 1135. Also, in this ST, different AMR profiles were found within one herd (profiles S and B were found on one farm and profiles B and C were found on another).

Three AMR profile variations were found in ST 316, ST 855, and ST 877. Two AMR profiles were determined in ST 876, ST 1437, and ST 1438. In genotype ST 877, two profiles of AMR were detected in the same herd (profiles B and F).

Multidrug-resistant profiles (B, I, K, L) were found in 22 isolates belonging to 22 different MLST genotypes. The most resistant strain (resistant to TET, STR, CLI, PIR, ERY) belonged to ST 1447 and it was hasA and hasB gene-negative (see Table 2).

### 3.5. Heterogeneity of S. uberis within a Herd

Nine isolates were detected in HERD-1, in which seven MLST genotypes and four AMR profiles (S, A, B, C) were determined including isolates fully susceptible, isolates resistant to a particular antibiotic, and multidrug-resistant isolates. In another farm (HERD-2), five isolates were detected and showed four MLST genotypes and three AMR profiles (A, B, E). In HERD-3, six isolates, in which there were five MLST genotypes and three AMR profiles (S, A, B), were determined; one of the isolates strongly adhered to the agar surface. In HERD-4, five isolates of four MLST genotypes were detected, which showed two AMR profiles (S, B), one that was susceptible and the other multidrug-resistant. HERD-5, HERD-6, and HERD-7 (each with three isolates) showed three MLST genotypes and three AMR profiles (S, A, B) (S, B, C) (A, B, H). Herd-8 (three isolates) showed three MLST genotypes and two AMR profiles (A, I). Herd-9 (three isolates) showed two MLST genotypes and three AMR profiles (S, B, D). In the other herds, only one or two isolates were detected.

### 3.6. Association of Genotypes with the Source of Samples

ST 1135 (nine isolates) was isolated predominantly from acute mastitis, subclinical mastitis, and healthy udders (see Table 3).

ST 307 (nine isolates) was isolated from acute mastitis, chronic recurrent mastitis, and udder surface swabs.

ST 1436 (four isolates) was isolated from acute mastitis, chronic recurrent mastitis, udder surface swabs, and healthy udders.

ST 316 (three isolates) was isolated from acute and subclinical mastitis and udder surface swabs.

ST 876 and ST 877 (each with three isolates) were isolated from acute mastitis.

ST 855 (three isolates) was isolated from acute and subclinical mastitis.

ST 1437 (three isolates) was isolated from acute mastitis and udder surface swabs.

ST 1438, ST 1439, ST 1440, and ST 1442 (each with two isolates) were isolated predominantly from acute mastitis and occasionally from subclinical mastitis.

ST 1441 (two isolates in one herd) was detected only in udder surface swabs and milk from healthy udders, whereas no isolate of this genotype was detected in mastitis milk.

## 4. Discussion

In this study, we assessed the heterogeneity of *S. uberis* isolates from 74 dairy farms obtained predominantly from subclinical, acute, and chronic recurrent mastitis, as well as from udder surface swabs and milk from healthy udders.

Out of 124 isolates, 89 MLST genotypes, 7 different virulence profiles, and 12 AMR profiles were identified. Only 23.6% of MLST allelic profiles could be assigned to known STs according to the database, indicating high heterogeneity of *S. uberis* strains worldwide. Similar results were also found in other studies, e.g., in the study by Silva et al. [25], in which 85.5% of the strains did not match with the known STs.

Some countries have relatively less heterogeneous *S. uberis* populations (UK, Portugal), whereas some others harbour more diverse populations of STs (Sweden, Switzerland) [11,12]. Some authors in their epidemiologic studies suggested that a more genetically diverse population of *S. uberis* in the herd assumes an environmental route of transmission and that high incidences of certain strains over others in a herd indicate contagious transmission [2,8]. Although this could be the case, it is considerably more likely that contagious transmission has resulted due to a fault in milking hygiene, permitting the transmission of bacteria from one mammary quarter to another. It could also be due to strains whose quantity in the environment exceeds the quantities of other strains and therefore the infection and re-infection of the mammary gland are much more likely. The very large number of different MLST allelic profiles in this study points to the huge heterogeneity of strains in dairy herds in the Czech Republic. In addition, many genotypes within the one herd were detected in many farms (up to seven MLST genotypes and four AMR profiles in one herd). This heterogeneic population structure might suggest that environmental transmission is the predominant route of infection in herds in the Czech Republic.

Out of 89 MLST genotypes, only 8 belonged to the known Global Clonal Complex (GCC): 7 STs (containing 27 isolates; 22%) belonged to GCC5, which has been identified as the major lineage among *S. uberis* isolates causing bovine mastitis in Europe [8,11], and 1 ST (containing 1 isolate; 0.8%) belonged to GCC143, which according to earlier reports is rare in Europe and is more often detected in Australia and India [11]. Other detected MLST genotypes, even if they belonged to known STs, did not belong to any GCC.

Rahman et al. [12] in their study evaluating the information about the strains available in the PubMLST database for *S. uberis* also indicated a high number of STs and showed that very few strains were shared between two countries and no particular strain showed worldwide prevalence. However, they also described strains that were shared between two countries from different continents such as ST 233 (shared among the UK, Canada, and Sweden) [12] or ST 60 and ST 184 (in Australia and Europe) [26]. In our study, ST 319 found in one isolate in one farm was also reported in Switzerland and Italy [11,12], ST 316 found in three isolates in three farms was detected in Switzerland [11], and ST 22 found in one isolate in one farm was also prevalent in the UK [8]; however, no other ST has been found in any other country [25,26,27,28].

In our study, ST 1135 and ST 307 were the most common types isolated from mastitis, but due to the huge heterogeneity of the isolated strains in our study, it was not possible to evaluate the association between the MLST genotype and the origin of the sample or the severity of mastitis. Some studies indicated the connection of some ST types or clonal complexes with a more serious course of infection and with the occurrence of clinical mastitis. For example, Käppeli et al. [11] described five causes of acute mastitis associated with ST 933 (CC5) in one herd in Switzerland. In Australian isolates, Tomita et al. [26] found that CC5 and CC143 were highly associated with clinical and subclinical mastitis and might represent a lineage of virulent isolates, whereas isolates belonging to CC86 were associated with low-cell-count cows. Furthermore, Rahman observed in his study based on the database that *S. uberis* CC5 and CC143 complexes were more prevalent in mastitis infections [12], but the same author also reported that no direct correlation existed between a type of ST complex and the severity of disease. Other examples of the lack of clear correlation between a particular strain type and infection level are ST-5 and ST-6 as they have been shown to be prevalent in clinical and subclinical mastitis infections as well as in healthy cattle [12]. Also in our study, isolates belonging to ST 1135 and isolates from ST 1436 were isolated from acute clinical and subclinical mastitis infection cases as well as from healthy mammary glands. Another example is the prevalent isolates from ST 307 and ST 1436, which were isolated from chronic recurrent mastitis, indicating the ability to persist in the mammary gland with possible contagious transmission, but these isolates were also collected from swabs of the udder surface, indicating an environmental mode of spread. In one herd, ST 1441 was detected only in the environment and in healthy udders and was not detected in mastitis milk. However, these are only two isolates of this genotype in one herd and therefore a possible connection with avirulence cannot be generalized.

Furthermore, due to the huge heterogeneity of the isolated strains, it was not possible to evaluate the prevalence of the virulence factors in certain MLST genotypes. However, it is clear from the results that isolates of certain MLST genotypes can possess a different set of virulence factor genes (ST 307 in our study showed two different virulence profiles) and thus can differ in their ability to cause mastitis or survive inside the mammary gland despite the immune reaction. On the other hand, a virulence gene or a specific set of virulence factor genes has not yet been identified, the presence or absence of which would clearly determine the virulence or avirulence of the strain, and also studies of comparative genomics of *S. uberis* did not reveal differences between the genome content of clinical and non-clinical strains [29]. Moreover, the clinical or non-clinical course of infection depends to a large extent on the condition of the mammary gland and the fitness of the animal, and it is not possible to unambiguously assess the virulence of a given isolate and to accurately categorize isolates as highly virulent and low-virulent.

Herein, 14 antimicrobials of 9 antibiotic classes were selected and tested with regard to their availability for intramammary treatment. Some of them are not intended or are not registered for the treatment of mastitis in the Czech Republic but are frequently used for the treatment of other bacterial diseases in farm animals so they were included for epidemiological purposes and to screen for their overall resistance to gain information about heterogeneity within MLST genotypes and in *S. uberis* within the same herd. In our study, we detected up to three different resistance profiles within a single MLST genotype. The results of our study showed that fully susceptible isolates coexisted with resistant or even multiresistant isolates in one herd, regardless of whether they belonged to the same MLST type.

The possession of virulence genes can vary widely geographically. In our study, we found a very high occurrence of *hasC*, *sua*, *gapC*, *oppF*, and *pauA/skc* genes (present in 97.6–100% isolates) and a lower occurrence of the *cfu* gene (8.9%). In some countries, the occurrence of virulence genes was similar [10], whereas in others, the situation was quite different. As an example of the different detection rates, the *oppF* gene was detected in almost 100% of isolates in Thailand, New York State, and the Czech Republic [3,30], but in the study of El-Aziz et al. [31] in Argentina, the *oppF* gene was detected in only 12% of the *S. uberis* isolates. The results of this study also showed a very different occurrence of the *pauA/skc* gene. Another example is the *cfu* gene, whose prevalence ranges from 4% in Germany [32] to 6% in our study, 19% in Poland [10], and up to 35% in Thailand [30] or 77% in Argentina [16]. These geographical differences again confirm the enormous heterogeneity of this pathogen in cow herds and the difficulty in determining the genes responsible for the development of mastitis.

## 5. Conclusions

It should be noted that MLST is an excellent tool for epidemiological studies, providing substantial discriminatory power for subtyping. In addition, its great advantage is the ability to compare results from different farms all over the world thanks to the available database. However, the determination of the MLST or ST type alone does not predict the degree of virulence of the isolate, its ability to survive in the mammary gland, or its resistance to antimicrobials because, according to our study, there is large heterogeneity even within a single MLST type. The genetic diversity of *S. uberis* is believed to be a barrier to the development of an effective vaccine and causes difficulties in implementing effective control measures. Many unknown questions remain to be clarified to understand the pathogenesis of *S. uberis* mastitis.

## Figures and Tables

**Table 1:** Oligonucleotide primers used in this study for *S. uberis* gene detection.

Virulence Factor	Genes	Nucleotide Sequence (5′-3′)	Amplicon Size	References
Hyaluronic acid	*hasA*	GAAAGGTCTGATGCTGATG	319	[15]
		TCATCCCCTATGCTTACAG		
Hyaluronic acid	*hasB*	TCTAGACGCCGATCAAGC	532	[15]
		TGAATTCCTATGCGTCGATC		
Hyaluronic acid	*hasC*	TGCTTGGTGACGATTTGATG	225	[15]
		GTCCAATGATAGCAAGGTCAC		
Epithelial cell invasion	*sua*	ACGCAAGGTGCTCAAGAGTT	776	[16]
		TGAACAAGCGATTCGTCAAG		
Surface dehydrogenase protein	*gapC*	GCTCCTGGTGGAGATGATGT	200	[16]
		GTCACCAGTGTAAGCGTGGA		
CAMP factor	*cfu*	TATCCCGATTTGCAGCCTAC	205	[16]
		CCTGGTCAACTTGTGCAACTG		
Solvent active transfer	*oppF*	GGCCTAACCAAAACGAAACA	419	[17]
		GGCTCTGGAATTGCTGAAAG		
Plasminogen activator	*pauA/skc*	TTCACTGCTGTTACATAACTTTGTG	976	[18]
		CCTTTGAAAGTGATGCTCGTG		
*S. uberis* specific	16S rRNA ub	CGCATGACAAT GGGTACA	445	[19]

**Table 2:** Characterisation of 124 *Streptococcus uberis* isolates—distribution of sequence types and allelic profiles, virulence profiles, resistance profiles, frequency of isolation.

ST ^a^	MLST Allelic Profile ^b^							GCC ^c^	No of Isolates	No of Farms	Virulence Profile ^d^	Resistance Profiles ^e^
1135	1	37	4	1	2	1	3	5	9	7	common	S, B, C
307	1	1	4	1	2	1	3	5	5	3	common	S, A, D
									3	2	cfu+	A
1436	1	1	1	1	1	1	3		4	3	common	A
316	2	1	4	1	2	1	3	5	3	3	common	S, A, E
855	9	1	27	2	39	1	3		3	3	common	S, D, E
876	1	1	4	1	65	1	3	5	3	3	common	S, A
877	2	1	4	2	2	1	3		3	2	common	S, B, F
1437	1	1	4	1	1	1	3		3	3	common	S, A
1438	1	1	4	2	29	1	3		2	2	common	S, H
1439	2	1	4	1	43	1	3		2	2	common	S
1440	1	1	43	1	43	1	3		2	2	common	S
1441	40	1	4	1	2	1	3		2	1	common	A
1442	2	1	27	2	39	4	3		2	1	common	S
22	2	1	2	1	2	1	2	5	1	1	common	A
63	1	1	5	1	2	1	3	5	1	1	common	S
308	40	1	4	2	49	1	3		1	1	common	S
319	5	15	5	2	2	1	3		1	1	cfu+	S
332	1	1	1	1	2	1	3		1	1	common	S
386	1	2	3	2	1	1	35		1	1	common	L
451	9	1	2	2	7	1	3	143	1	1	cfu+	S
501	1	1	4	2	49	1	3		1	1	common	A
877	2	1	4	2	2	1	3		1	1	common	H
878	2	1	4	1	76	1	3		1	1	common	E
884	8	1	6	4	3	2	3		1	1	common	S
895	5	42	5	2	2	3	3		1	1	hasA−, hasB−	S
914	2	1	4	1	65	1	3		1	1	common	G
1065	2	1	5	1	2	1	3	5	1	1	common	B
1127	2	1	5	1	65	1	3		1	1	common	S
1204	3	6	5	2	10	4	10		1	1	hasA−, hasB−	S
1443	1	4	4	1	5	2	3		1	1	hasA−, hasB−	S
1444	2	2	5	2	3	4	3		1	1	hasA−, hasB−, cfu+	A
1445	3	1	41	4	5	2	10		1	1	hasA−, hasB−	S
1446	3	25	29	2	5	2	3		1	1	hasA−, hasB−	S
1447	9	1	5	2	29	2	3		1	1	hasA−, hasB−	I
1448	21	2	5	2	3	4	9		1	1	hasA−, hasB−, cfu+	A
1449	42	30	4	1	70	4	3		1	1	hasA−, hasB−, cfu+, pauA/skc−	L
1450	42	2	4	22	65	4	10		1	1	cfu+	A
1451	42	30	4	2	70	4	15		1	1	hasA−, hasB−, cfu+, pauA/skc−	H
1452	42	64	4	2	70	4	15		1	1	hasA−, hasB−, pauA/skc-	S
1453	55	30	4	1	88	4	3		1	1	cfu+	K
All other allelic profiles were detected only in one isolate and showed a common virulence profile.												

^a^ ST = sequence type. ^b^ Multilocus Sequence Typing (MLST) allelic profile with the following order: *arcC, ddl, gki, recP, tdk, tpi, ygiL*. ^c^ GCC = Global Clonal Complex for each ST has been assigned by the *S. uberis* MLST database. Empty field means that it does not belong to any GCC. ^d^ Common profile = hasA+, hasB+, hasC+, sua+, cfu-, pauA/skc+, gapC+, oppF+; differences from the common profile are marked in green. ^e^ Different resistance profiles are marked with letters for better clarity in the table; **S** = susceptible to all tested antimicrobials; **A** = resistant to TET; **B** = resistant to TET, STR, CLI; **C** = resistant to TET, STR; **D** = resistant to STR, CLI; **E** = resistant to STR, CLI, PIR; **F** = resistant to STR, RIF; **G** = resistant to TET, CLI; **H** = resistant to TET, ERY; **I** = resistant to TET, STR, CLI, PIR, ERY; **J** = resistant to STR; **K** = resistant to TET, CLI, ERY; **L** = resistant to TET, CLI, PIR, ERY. Antimicrobial abbreviations: TET–tetracycline; STR–streptomycin; CLI–clindamycin; PIR–pirlimycin; RIF–rifampicin; ERY–erythromycin. Multiresistant profiles are marked in red.

**Table 3:** Source of isolation of *S. uberis* sequence types (STs) ^a^.

Sequence Type	No of Isolates	Source				
		Acute Mastitis	Subclinical Mastitis	Chronic Mastitis	Healthy Udder	Udder Surface Swabs
ST 1135	9	6	2	0	1	0
ST 307	9	4	0	3	0	2
ST 1436	4	1	0	1	1	1
ST 316	3	1	1	0	0	1
ST 876	3	3	0	0	0	0
ST 877	3	3	0	0	0	0
ST 855	3	1	2	0	0	0
ST 1437	3	2	0	0	0	1
ST 1438	2	2	0	0	0	0
ST 1439	2	1	1	0	0	0
ST 1440	2	2	0	0	0	0
ST 1442	2	2	0	0	0	0
ST 1441	2	0	0	0	1	1

^a^ For each ST, the number of isolates and their source is shown. Only STs that occurred more than once are listed in the table.

## Data Availability

The data are available on request from the corresponding author. The more detailed data about farms are not publicly available to protect the privacy of farm owners.
